# Sex dependent risk factors for mortality after myocardial infarction: individual patient data meta-analysis

**DOI:** 10.1186/s12916-014-0242-y

**Published:** 2014-12-17

**Authors:** Hanna M van Loo, Edwin R van den Heuvel, Robert A Schoevers, Matteo Anselmino, Robert M Carney, Johan Denollet, Frank Doyle, Kenneth E Freedland, Sherry L Grace, Seyed H Hosseini, Kapil Parakh, Louise Pilote, Chiara Rafanelli, Annelieke M Roest, Hiroshi Sato, Richard P Steeds, Ronald C Kessler, Peter de Jonge

**Affiliations:** Interdisciplinary Center Psychopathology and Emotion Regulation (ICPE), Department of Psychiatry, University of Groningen, University Medical Center Groningen, Hanzeplein 1, PO box 30.001, 9700 RB Groningen, The Netherlands; Department of Mathematics and Computer Science, Eindhoven University of Technology, Den Dolech 2, 5612 AZ Eindhoven, The Netherlands; Division of Cardiology, Department of Medical Sciences, Città della Salute e della Scienza, University of Turin, C.so A.M. Dogliotti, 14, 10126 Turin, Italy; Department of Psychiatry, Washington University School of Medicine, 4320 Forest Park Avenue, St. Louis, Missouri 63108 USA; CoRPS-Center of Research on Psychology in Somatic diseases, Tilburg University, Warandelaan 2, 5000 LE Tilburg, The Netherlands; Division of Population Health Sciences (Psychology), Royal College of Surgeons in Ireland, 123 St Stephen’s Green, Dublin 2, Ireland; Faculty of Health, York University and University Health Network, 368 Norman Bethune, 4700 Keele Street, Toronto, M3J 1P3 Canada; Psychiatry and Behavioral Sciences Research Center, Addiction Institute, Mazandaran University of Medical Sciences, Psychosomatic department, Imam hospital, Sari, Iran; John Hopkins School of Medicine, John Hopkins Bloomberg School of Public Health, John Hoplins Bayview Medical Center, 4940 Eastern Avenue, Baltimore, Maryland 21224 USA; Division of General Internal Medicine, McGill University, McGill University Health Centre, 687 Pine Avenue West, V Building, V2.17, Montreal, H3A 1A1 Canada; Department of Psychology, University of Bologna, Viale Berti Pichat 5, 40127 Bologna, Italy; School of Human Welfare Studies, Kwansei Gakuin University, 1-1-155, Uegahara, Nishinomiya, Hyogo 662-8501 Japan; Department of Cardiology, Queen Elizabeth Hospital, Edgbaston, Birmingham, B15 2TH West Midlands UK; Department of Health Care Policy, Harvard Medical School, 180 Longwood Avenue, Boston, Massachusetts 02115 USA

**Keywords:** All-cause mortality, Interactions, Myocardial infarction, Prediction, Risk factors, Sex

## Abstract

**Background:**

Although a number of risk factors are known to predict mortality within the first years after myocardial infarction, little is known about interactions between risk factors, whereas these could contribute to accurate differentiation of patients with higher and lower risk for mortality. This study explored the effect of interactions of risk factors on all-cause mortality in patients with myocardial infarction based on individual patient data meta-analysis.

**Methods:**

Prospective data for 10,512 patients hospitalized for myocardial infarction were derived from 16 observational studies (MINDMAPS). Baseline measures included a broad set of risk factors for mortality such as age, sex, heart failure, diabetes, depression, and smoking. All two-way and three-way interactions of these risk factors were included in Lasso regression analyses to predict time-to-event related all-cause mortality. The effect of selected interactions was investigated with multilevel Cox regression models.

**Results:**

Lasso regression selected five two-way interactions, of which four included sex. The addition of these interactions to multilevel Cox models suggested differential risk patterns for males and females. Younger women (age <50) had a higher risk for all-cause mortality than men in the same age group (HR 0.7 vs. 0.4), while men had a higher risk than women if they had depression (HR 1.4 vs. 1.1) or a low left ventricular ejection fraction (HR 1.7 vs. 1.3). Predictive accuracy of the Cox model was better for men than for women (area under the curves: 0.770 vs. 0.754).

**Conclusions:**

Interactions of well-known risk factors for all-cause mortality after myocardial infarction suggested important sex differences. This study gives rise to a further exploration of prediction models to improve risk assessment for men and women after myocardial infarction.

**Electronic supplementary material:**

The online version of this article (doi:10.1186/s12916-014-0242-y) contains supplementary material, which is available to authorized users.

## Background

About 8% of patients die within the first months after myocardial infarction, whereas approximately 30% survive more than 20 years [1,2]. Several risk assessment instruments have been developed to quantify the risk of mortality in individual patients, such as the Global Registry of Acute Coronary Events and Thrombolysis in Myocardial Infarction risk scores [3-5]. These instruments are based on a set of well-known risk factors for mortality such as age, heart failure, and comorbidity. Unfortunately, interactions of these risk factors are rarely investigated optimally, while these could be of relevance in accurately differentiating the high risk from the low risk patients.

Some interactions of this type have been identified, such as between sex and age and between sex and left ventricular ejection fraction (LVEF), in predicting mortality after myocardial infarction [2,6-8]. These interactions suggested that mortality after myocardial infarction is higher in young women than in young men, while poor LVEF was associated with an increased risk of death, especially in men. However, previous studies have only investigated interactions sporadically and did not perform systematic searches through all or many interactions in a sufficiently large dataset to estimate interaction effects reliably. To discover currently unknown interactions of interest, we systematically investigated a large set of interactions that potentially influence prediction of all-cause mortality in patients with myocardial infarction with a novel combination of statistical learning methods [9,10].

## Methods

### Data

Data was retrieved from the MINDMAPS dataset, an individual patient data meta-analysis dataset that combines information from 16 observational studies on baseline predictors of subsequent all-cause mortality among 10,512 patients with myocardial infarction [11]. All 16 studies began with samples of patients hospitalized for myocardial infarction according to standardized diagnostic criteria, of whom survival was assessed during follow-up. The included studies were performed in nine different countries: ten studies in Europe (Ireland, Italy, The Netherlands, Sweden, United Kingdom), four in North America (Canada, United States of America), one in Asia (Japan), and one in the Middle East (Iran). For a detailed description of the review process and study characteristics, we refer to Meijer et al. [11]. All individual studies obtained ethical approval and participants provided written informed consent [11].

### Measures

#### Predictors

The predictors included those considered in previous studies of risk factors for mortality after myocardial infarction: severity of the heart disease, general health, comorbidity, sex, and age [3,5,11]. Severity of heart disease was indicated by history of myocardial infarction, LVEF, clinical signs of heart failure (Killip class), and beta-blocker use. Beta-blocker use was selected as a measure of cardiovascular medication use because information on this drug was most often available. History of myocardial infarction and beta-blocker use were dichotomized into ‘yes’ and ‘no’; LVEF was dichotomized into significant left ventricular dysfunction present (<40%) and absent (≥40%); Killip class was dichotomized into no clinical signs of heart failure (class I) and clinical signs of heart failure (class II, III, IV) as the four-category scores were not available in all studies. Measures of general health were smoking, hyperlipidaemia, and body mass index (BMI). Smoking was dichotomized into ever (current or previous) versus never smoking. BMI was categorized into three classes: low (BMI <20), intermediate (BMI 20–30), and high (BMI >30). Comorbid diabetes mellitus, depression, and use of antidepressants were also included as predictors. The latter variables were included because of evidence from previous meta-analyses that depression independently predicts all-cause mortality in patients with coronary disease and myocardial infarction [11,12]. The level of depression was measured as a total score on either self-report questionnaires (mostly the Beck Depression Inventory (BDI), or the Zung Self-rating Depression Scale (ZSDS)) or standardized structured diagnostic interviews (such as the Composite International Diagnostic Interview, or the Structured Clinical Interview for DSM Disorders) within three months after myocardial infarction. For a detailed description of questionnaires used in the different studies, we refer to Meijer et al. [11]. Total depression scores were standardized into *z*-scores within each study in order to account for the different questionnaires. These depression *z*-scores were categorized into three classes: low (*z*-score in lowest quartile), intermediate (*z-*scores in intermediate quartiles), and high (*z*-score in highest quartile). Finally, the demographic variables sex and age were included in the set of predictors. Age was categorized into low (<50 years), intermediate (50–70 years), and high (>70 years).

The continuous variables depression *z*-score, BMI, and age were categorized into three levels to enable the discovery of possible non-linear associations between these predictors and all-cause mortality. Based on previous studies, we expected that patients with an extreme low or high BMI, age, and depression score could potentially have a higher risk of mortality, as opposed to patients with an average score on these variables [13] or that there could be interactions with sex for extreme values of these variables, but not for average values [6,7]. Categorization of these risk factors into a low, intermediate, and high level enabled tracing such potential non-linear relationships. The intermediate classes were taken as reference classes, as they represented the largest group of patients seen in clinical practice. All logically possible two-way and three-way interactions of the predictors were included to enable a systematic data-driven investigation of interactions.

#### Outcome

All-cause mortality was chosen as primary outcome; time-to-event data were used.

### Imputation

The extent of missing data varied across studies and some variables were not assessed in some studies. On average, 18.0% of values were missing. After pooling all studies, we imputed those values in one data set using the Multivariate Imputation by Chained Equations algorithm in R with predictive mean matching for continuous variables and logistic regression for binary variables (20 iterations, R package *mice*, version 2.21) [14]. To account for possible systematic differences in the individual studies, we included the variable ‘study’ as a factor in the imputation. Survival data of cardiovascular events were included as extra predictors in the imputation to improve the imputation results, in addition to survival data of all-cause mortality and all predictors mentioned above [15].

### Statistical analyses

Data were randomly divided into an 80% training sample (n = 8,410) and a 20% validation sample (n = 2,102). The training sample was used for model discovery with 10-fold cross-validation, and the independent validation sample was used to assess the predictive performance of the models. All analyses were performed in R [16].

#### Lasso regression analyses to identify interactions

Least Absolute Shrinkage and Selection Operator (Lasso)-penalized Cox regression was used to trace two-way and three-way interactions in addition to main effects. Penalized linear regression methods include a penalty for model complexity such as the Lasso [9]. This penalty constrains the sum of the absolute values of the regression coefficients, consequently shrinking the regression coefficients and selecting a limited set of predictors. The method is developed to increase prediction accuracy by diminishing variance or overfitting (which occurs when the model follows noise in the data too closely) in situations with many predictors. These features make Lasso regression well-suited for a situation like the current one in which a large set of interactions is analyzed and not all of them will be related to the outcome [10]. The method has been used before in other studies to select interactions in high dimensional analyses of genome-wide association studies [17].

We used the Lasso implementation for Cox regression *glmnet* in R*,* which returns a range of more to less extensive regression models dependent on the size of the Lasso penalty (version 1.9-5) [18,19]. Predictors included all main effects and 518 two-way and three-way interactions. Standardization of coefficients was not needed as all predictors were binary (dummy) variables. We selected the Lasso model that resulted in minimal prediction error in 10-fold cross-validation in the 80% training sample (n = 8,410). Prediction error for Cox Lasso models was measured in terms of partial likelihood deviance [19]. From the Lasso model with least prediction error, we selected interactions with penalized beta coefficients ≥0.1 or ≤ −0.1 to retain the most relevant parameters. Consistency of the Lasso results was checked by repeating the same procedure 100 times in 80% random draws of the training sample (n = 6,728). The number of times that specific interactions were selected in those 100 Lasso regression models with beta-coefficients ≥0.1 or ≤ −0.1 was assessed and compared to the model on the full training sample (n = 8,410).

#### Multilevel Cox models

Multivariate multilevel Cox regression analyses were used to estimate the unpenalized effect sizes of risk factors and interactions (R-package *coxme*, version 2.2-3, Breslow ties) [20]. The proportional hazards assumption for Cox regression was met [11]. Similar to the study of Meijer et al. [11], we included the individual studies as separate levels by adding variable ‘study’ as a random effect in the models. This was done to account for systematic differences between studies and thus the expectation that different effect sizes could be underlying the different studies [21,22]. The multilevel Cox models were built with data of the training sample (n = 8,410). First, we studied the model with main effects only. Subsequently, we checked the statistical significance of the interactions selected by the Lasso by including each to the main effects model one at a time. Stratified analyses were performed to estimate differences of effect sizes of risk factors for different subgroups of the sample in case of significant interactions.

#### Test model performance in independent validation sample

To account for overfitting, the predictive performance of the Cox regression model was determined in the independent 20% validation sample (n = 2,102) and compared to the performance in the 80% training sample (n = 8,410). Prediction accuracy was measured by assessing the areas under the receiving operating characteristic-curve (AUC) for predicting mortality at 3 years after myocardial infarction (R package *survivalROC*, version 1.0-3, Kaplan Meier method) [23]. Bootstrapping was used to calculate confidence intervals of the AUC’s (R package *boot*, version 1.3-13, 1,000 bootstrapped samples, percentile bootstrap) [24].

## Results

### Patient characteristics

Individual patient data from each study were combined, resulting in a sample of 10,512 patients with myocardial infarction. Since we used imputation in the current study, we included 337 subjects, which is more than in the study of Meijer et al. [11]. The mean age of the sample was 61 (SD 11.9 years), 71% of the patients were male, and the mean BMI was 27 kg/m^2^ (Table 1). Concerning the cardiac disease severity at baseline: 19% of the patients had a history of myocardial infarction prior to the index episode, 18% showed clinical signs of heart failure (Killip class II–IV), and 23% had a low LVEF (<40%). On average, 21% of the patients had comorbid diabetes, and 40% had an elevated depression score as assessed by structured diagnostic interviews and standard cut-off values on self-report questionnaires (such as a BDI-1A ≥10, or a ZSDS ≥40; for a full overview see Meijer et al. [11]). Patients with elevated depression scores might be overrepresented as some studies have oversampled patients with depression [11]. Subjects in the highest quartile of depression *z*-scores had a mean score on the BDI-1A of 21.0 and 10.7% used antidepressants, as opposed to 4.9% in the group with lower depression *z*-scores. The mean follow-up time was 3.6 years (median 3.0 years). Maximum follow-up time varied across studies between 1.0 and 8.2 years. In total, 14% of the patients died during follow-up.

**Table 1:** **Baseline characteristics**

	Mean (s.d.)/%*
**Demographics**	
Age (years)	61.0 (11.9)
Male	70.9
**Heart disease**	
History of MI	18.8
LVEF <40%	23.0
Killip class II–IV	18.1
Beta-blocker use	72.0
**Comorbidity**	
Diabetes	21.3
Elevated depression score^†^	39.7
BDI in highest quartile depression *z*-score^‡^	21.0 (7.7)
BDI in intermediate quartiles depression *z*-score^‡^	8.8 (4.3)
BDI in lowest quartile depression *z*-score^‡^	2.3 (2.1)
Antidepressant use	6.5
**General health**	
BMI (kg/m^2^)	27.0 (5.0)
Smoking (ever)	44.7
Hyperlipidaemia	46.9

BDI, Beck Depression Inventory version; BMI, Body mass index; LVEF, Left ventricular ejection fraction; MI, Myocardial infarction.

*Means of age, BDI-scores and BMI, percentages otherwise of original data. Differences in baseline characteristics with respect to Meijer et al. [11] are due to the fact we included 377 patients more as we used imputation of missing values for depression.

^†^Elevated depression score as assessed by structured diagnostic interviews and standard cut-off values on self-report questionnaires.

^‡^Mean BDI-1A scores for subjects in highest, intermediate and lowest quartile of depression *z*-scores for n = 6,423 subjects in which BDI-1A scores were measured.

### Lasso regression: selection of interactions

Eight main effects and seven two-way interactions were found to be stable predictors of mortality with penalized beta coefficients ≥0.1 or ≤ −0.1 in Lasso regression analysis of the full training sample (n = 8,410, Table 2). The main effects older age (>70 years), a history of myocardial infarction, and clinical signs of heart failure (Killip class II–IV) strongly predicted all-cause mortality. Also a poor LVEF, comorbid diabetes, and a low BMI were associated with a higher risk of all-cause mortality. Instead, a younger age (<50 years) and beta-blocker use were protective. Four of the selected interactions concerned sex, in combination with a younger age (<50 years), high depression score, low LVEF (<40%), and hyperlipidaemia. Two of the selected interactions were a combination of beta-blocker use and clinical signs of heart failure (Killip class II–IV) or diabetes. The last interaction consisted of a combination of heart failure and hyperlipidaemia. No three-way interactions had sufficiently large effect sizes to be selected.

**Table 2:** **Selected main effects and interactions in Lasso regression analysis**

	β*	**Frequency** ^**†**^
**Demographics**		
Age <50	–0.47	
Age >70	0.86	
**Heart disease**		
History of MI	0.52	
LVEF <40%	0.31	
Killip class II–IV	0.54	
Beta-blocker use	–0.53	
**Comorbidity**		
Diabetes	0.47	
**General health**		
BMI <20	0.20	
**Selected interactions**		
Killip class * beta-blocker use	0.26	95/100
Male sex * LVEF <40%	0.14	68/100
Male sex * age <50	–0.18	64/100
Male sex * depression high^‡^	0.13	64/100
Male sex * hyperlipidaemia	–0.17	61/100
Diabetes * beta-blocker use	0.10	36/100
Killip class * hyperlipidaemia	0.10	34/100

β, Penalized beta-coefficient; BMI, Body mass index; LVEF, Left ventricular ejection fraction; MI, Myocardial infarction.

*All main effects and interactions with a penalized beta-coefficient ≥0.1 or ≤ –0.1 selected in Lasso regression analysis in the training data (n = 8,410).

^†^The number of times this interaction was found with a beta-coefficient ≥0.1 or ≤ –0.1 in 100 Lasso regression analyses in random 80% samples of the training data (n = 6,728).

^‡^Depression high: depression *z*-score in highest quartile.

Five of the selected interactions were observed >50 times in the 100 Lasso analyses on different 80% random subsamples of the training data which were performed to check the consistency of the results. These five concerned the four interactions including sex and the interaction of beta-blocker use and heart failure (Killip class II–IV). No other interactions were selected more than 50 times in the 100 Lasso runs (Additional file 1: Table S1). The five interactions that were observed more than 50 times were selected for inclusion in the multilevel Cox regression models.

### Cox regression: effect of interactions

Nearly all predictors were significantly associated with all-cause mortality in the unstratified multilevel Cox model with main effects only (*P* values <0.05; Table 3). Risk factors with the largest effect sizes were an older age (>70 years), diabetes, and all measures of heart disease: clinical signs of heart failure, history of myocardial infarction, and a low LVEF (hazard ratio (HR) range 1.6 to 2.8). Less strong, but also significantly predicting all-cause mortality, were a low BMI (<20 kg/m^2^), high depression score, and use of antidepressants (all HRs of 1.3). Significant protective factors were a younger age (<50 years) and beta-blocker use (HR’s of 0.5 and 0.8, respectively). Further, a depression score in the lowest quartile predicted lower mortality (HR 0.9), which indicated that depression scores were linearly associated with mortality, as opposed to predicting mortality only from a certain cut-off. Not significantly related to all-cause mortality were sex, smoking, a high BMI (>30 kg/m^2^), and hyperlipidaemia. The predictive accuracy of the model in the validation sample was fair (AUC 0.770). As expected, the model was more accurate in the training sample (AUC 0.812). Because the selected interactions mainly indicated sex differences, we tested the predictive accuracy of the model for men and women separately in post-hoc analysis of the validation data. The model was more accurate for men (AUC 0.770) than for women (AUC 0.754).

**Table 3:** **Effect sizes of risk factors in unstratified multilevel Cox model**

	HR*	**95% CI**	***P***
**Demographics**			
Male sex	1.0	(0.9–1.1)	0.86
Age <50	0.5	(0.4–0.6)	<0.001
Age >70	2.7	(2.4–3.1)	<0.001
**Heart disease**			
History of MI	1.8	(1.5–2.0)	<0.001
LVEF <40%	1.6	(1.4–1.8)	<0.001
Killip class II–IV	2.1	(1.8–2.3)	<0.001
Beta-blocker use	0.8	(0.7–0.9)	<0.001
**Comorbidity**			
Diabetes	1.8	(1.6–2.0)	<0.001
Depression low^†^	0.9	(0.7–1.0)	0.04
Depression high^†^	1.3	(1.1–1.4)	<0.001
Antidepressant use	1.3	(1.1–1.6)	0.01
**General health**			
Hyperlipidaemia	0.9	(0.8–1.0)	0.08
Smoking	1.1	(1.0–1.2)	0.22
BMI <20	1.3	(1.0–1.7)	0.03
BMI >30	0.9	(0.8–1.0)	0.09
**Predictive accuracy**			
AUC (general)^‡^	0.770 (0.730–0.809)		
AUC (male – female)^§^	0.770 (0.714–0.825) – 0.754 (0.687–0.816)		

AUC, Area under the curve; BMI, Body mass index; 95% CI, 95% Confidence interval; HR, Hazard ratio; LVEF, Left ventricular ejection fraction; MI, Myocardial infarction; *P*, *P* value.

*HR’s, 95% CI, *P* values and predictive accuracy for multivariate multilevel Cox models with main effects only in the training data (n = 8,410).

^†^Depression low and high: depression *z*-score in the lowest and highest quartile.

^‡^AUC in the validation data (n = 2,102).

^§^AUC for male patients (n = 1,475) and female patients (n = 627) in the validation data.

Evaluation of the five selected interactions with multilevel Cox model showed that some risk factors had different effect sizes in specific subgroups of patients with myocardial infarction and indicated sex differences in particular. Four out of five Lasso-selected interactions were significant or borderline significant when they were added to the unstratified multilevel Cox model one at a time (*P* values 0.02 to 0.08; Table 4). The only selected, non-significant interaction was sex in combination with hyperlipidaemia. The remaining sex-related two-way interactions, which were not selected by Lasso, were also not significant (*P* values 0.17 to 0.99). With sex-stratified analysis, we investigated the differential effect sizes of risk factors for men and women as suggested by the sex-related interactions. The interaction between sex and age <50 years suggested that, for women, a young age was less protective than for men (HR of 0.7 vs. 0.4, respectively, as compared to the reference class of 50 to 70 years; Additional file 2: Figure S1). Second, the interaction between sex and LVEF <40% suggested that a low LVEF was a stronger predictor of all-cause mortality in men (HR 1.7) than in women (HR 1.3; Additional file 3: Figure S2). Third, the interaction between sex and high depression score suggested that a high depression score increased the risk of all-cause mortality in men (HR 1.4), while it did not for women (HR 1.1; Additional file 4: Figure S4). Thus, young age, poor LVEF, and high depression had different effect sizes for men and women, but the direction of the effects were similar in both sexes (as opposed to increasing risk for men and decreasing risk for women or vice versa). A second stratified analysis was performed to investigate the interaction most often selected by Lasso, which was between beta-blocker use and clinical signs of heart failure (Killip class II–IV). This interaction was significant and suggested that, for patients without heart failure, beta-blocker use was more protective than for patients with heart failure (HR’s of 0.7 and 0.9, respectively, *P* value 0.016; Additional file 5: Figure S4).

**Table 4:** **Sex-stratified multilevel Cox models**

	**Female**			**Male**			**Inter.**
	HR*	**95% CI**	***P***	HR*	**95% CI**	***P***	***P*** ^**†**^
**Demographics**							
Age <50	0.7	(0.4–1.1)	0.087	0.4	(0.3–0.6)	<0.001	0.076^‡^
Age >70	2.5	(2.0–3.1)	<0.001	2.9	(2.5–3.3)	<0.001	0.17
**Heart disease**							
History of MI	1.8	(1.4–2.3)	<0.001	1.7	(1.5–2.0)	<0.001	0.99
LVEF <40%	1.3	(1.1–1.6)	0.018	1.7	(1.5–2.0)	<0.001	0.018^‡^
Killip class II–IV	2.1	(1.7–2.6)	<0.001	2.1	(1.8–2.4)	<0.001	0.41
Beta-blocker use	0.7	(0.6–0.9)	0.001	0.8	(0.7–0.9)	0.001	0.72
**Comorbidity**							
Diabetes	1.8	(1.4–2.2)	<0.001	1.7	(1.5–2.0)	<0.001	0.89
Depression low	0.8	(0.6–1.1)	0.27	0.9	(0.7–1.0)	0.13	0.67
Depression high	1.1	(0.9–1.4)	0.41	1.4	(1.2–1.6)	<0.001	0.054^‡^
Antidepressant use	1.2	(0.8–1.6)	0.37	1.3	(1.1–1.7)	0.017	0.36
**General health**							
Hyperlipidaemia	1.0	(0.8–1.2)	1.0	0.8	(0.7–1.0)	0.037	0.26^‡^
Smoking	0.9	(0.7–1.2)	0.54	1.2	(1.0–1.4)	0.068	0.48
BMI <20	1.4	(1.0–2.1)	0.071	1.3	(0.9–1.8)	0.14	0.91
BMI >30	0.9	(0.7–1.1)	0.33	0.9	(0.7–1.1)	0.19	0.64

BMI, Body mass index; CI, Confidence interval; HR, Hazard ratio; Inter., Interaction; LVEF, Left ventricular ejection fraction; MI, Myocardial infarction; *P*, *P* value.

*HR’s, CI’s and *P* values for sex-stratified multivariate multilevel Cox models in the training data (n = 8,410).

^†^ *P* value of two-way interactions between sex and all other risk factors based on addition of the interaction to the unstratified multilevel Cox model (such as in Table 3) one at the time.

^‡^ *P* value of the sex-related interactions selected by Lasso.

### Incidence rates

**Figure 1:**
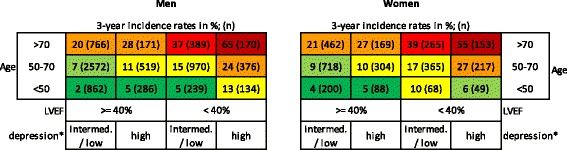
**Incidence rates of all-cause mortality in relation to sex, age, LVEF, and depression.** Abbreviation: LVEF, left ventricular ejection fraction. * Depression high: depression z-score in the highest quartile. Depression intermed./low: depression z-score in the lower three quartiles.

As most interactions involved sex, we calculated, in post-hoc analysis, the incidence rates of all-cause mortality in the 3 years after myocardial infarction among men and women in relation to age, depression score, and LVEF in the entire dataset (n = 10,512). In general, women had a higher risk of dying than men (3-year incidence rates of 17.0% vs. 12.0%). Nevertheless, men were more at risk of dying than women in the presence of certain combinations of risk factors. Most notable, the 3-year incidence rate of mortality for men was 65% when they were older than 70 years and had a high depression score and a low LVEF as compared to 55% for women with the same risk profile. Differences in 3-year incidence rates of all-cause mortality were more pronounced in men (min. 2% – max. 65%), than in women (min. 4% – max. 55%; Figure 1).

## Discussion

With a systematic data-driven search through all possible two-way and three-way interactions of risk factors for all-cause mortality after myocardial infarction, we found that some risk factors act differently in men and women. A high depression score was associated with increased mortality risk in men, but not in women. In addition, for women, a younger age was less protective than for men; LVEF <40% was more strongly predicting mortality in men than in women. Another main finding was that, in patients without heart failure, beta-blocker use was more protective than expected.

These results are limited in several ways. First, the data came from studies carried out between 1985 and 2006. As the group of patients and treatment of myocardial infarction are changing over time (more older patients, more female patients, and more comorbidity) [1,25,26], we should be cautious in generalizing the results to current patients with myocardial infarction. Indeed, replication is needed with newer data. Second, we could not include all relevant predictors, such as blood pressure, heart rate, waist circumference, kidney function, electrocardiogram findings, or educational attainment, marital status, and socioeconomic status, in our analyses as these data were not available in all studies [3-5,27,28]. Inclusion of such measures could have led to more precise risk assessment and possibly lead to other interaction patterns.

Within the context of these limitations, this is the first study performing a systematic search through a variety of interactions of risk factors for all-cause mortality after myocardial infarction using a large multi-study international sample. We applied statistical learning techniques to prevent overfitting, and tested the prediction of the identified model in an independent validation sample. Furthermore, selected interactions were clinically relevant as they indicated the importance of sex in differentiating patients with high and low risk for all-cause mortality after myocardial infarction.

Some interactions that were identified were congruent with previous studies, while others represented novel associations. Certain interactions, including sex, have been reported before such as the interaction between sex and age indicating that young women are at higher risk of all-cause mortality than young men [6,7,29]. Likewise, the interaction between sex and LVEF – suggesting that a low LVEF is a more important risk factor for men than for women – was consistent with findings in patients with myocardial infarction [8] and heart failure [30]. Two studies that focused on the interaction between sex and depression did not find substantial sex differences with regard to depression as opposed to our study, but the former studies included a relatively low number of women (n = 283 and n = 155, respectively) and used major cardiac events or cardiac mortality as outcome measures rather than all-cause mortality [8,31]. Finally, it has been previously shown that beta-blocker use is more protective after myocardial infarction in the absence of clinical signs of heart failure (Killip class I) than in the presence of these signs (Killip classes II–III) [32], which is in agreement with the findings in this study.

Our study demonstrates the potential importance of interactions in risk assessment in medicine. If interaction effects are not taken into account, statistical models will return an average estimate of the effect of risk factors in the entire patient population (e.g., depression increases risk of all-cause mortality). Instead, interactions allow for possible differences within the patient population and show that a risk factor might have a different effect in the presence of another risk factor (e.g., depression increases risk of all-cause mortality in men but not in women). In our study, interactions repeatedly suggested sex differences. Although baseline differences between men and women with myocardial infarction are declining, there are still substantial dissimilarities. Men have a higher risk of myocardial infarction, but women have a higher risk of death following myocardial infarction. Women presenting with myocardial infarction are generally older, have more comorbidities such as diabetes, hypertension, and heart failure, increasing the risk of all-cause mortality as compared to men [2,33-35]. All these differences could explain why different risk factors might differentially predict all-cause mortality for men and women.

The results reported here suggest three broad classes of extensions in further research. First, follow-up studies could focus on additional interactions of interest in order to obtain more specific risk assessment for subgroups of patients with myocardial infarction. The investigated set of interactions in this study was not comprehensive. Moreover, as Lasso tends to leave out correlated interactions, there could be interactions of interest that we did not identify in this study such as diabetes and depression [36], smoking and sex [8], and LVEF and Killip class [37]. In addition, it would be worth looking at interactions including other well-known risk factors of all-cause mortality after myocardial infarction such as cardiac arrest at admission, family history, and anxiety disorders [5,38,39]. Second, more research is needed to identify mechanisms underlying the identified interactions. For instance, why is depression more strongly related to all-cause mortality in men than in women? A study in one of the datasets included in MINDMAPS found that part of this interaction effect can be explained by the fact that men with depression are more likely to have a poor LVEF than women with depression [40]. Depression reflected more severe heart disease in men but not in women, which partly explains why depressed men are more at risk for all-cause mortality than depressed women. Other confounders could underlie the remaining interaction effect between sex and depression found in this study after controlling for severity of heart disease. Thus, the interpretation of interactions should be done in the context of possible confounders. The finding that smoking did not have a significant main effect in this study should also be seen in this light. In addition to a broad set of important risk factors, such as history of myocardial infarction, heart failure, age, and comorbidity, smoking did not significantly predict all-cause mortality risk, but this does not imply that smoking on itself does not increase risk of all-cause mortality after myocardial infarction.

Finally, the interactions including sex lead to doubt on the performance of general risk assessment instruments for both sexes: are they equally accurate for men and women? Future studies could explore if different prediction algorithms for men and women would increase prediction accuracy, and if this benefit would outweigh the complexity for clinical practice of working with two different instruments instead of one.

## Conclusions

Interactions might increase understanding and prediction of all-cause mortality after myocardial infarction. Our key finding was that marked sex differences exist, indicating that future research is warranted in order to improve risk assessment for both men and women with myocardial infarction.

## Additional files

Additional file 1: Table S1. Selected interactions in 100 Lasso regression analyses. Additional file 2: Figure S1. Survival by sex and age. Survival curves of all subjects (n = 10,512) stratified by sex and age (in years) adjusted for all other risk factors. M, Male; F, Female. Additional file 3: Figure S2. Survival by sex and left ventricular ejection fraction (LVEF). Survival curves of all subjects (n = 10,512) stratified by sex and LVEF adjusted for all other risk factors. M, Male; F, Female; LVEF, Left ventricular ejection fraction. Additional file 4: Figure S3. Survival by sex and depression. Survival curves of all subjects (n = 10,512) stratified by sex and depression *z*-score adjusted for all other risk factors. High depression: depression *z-*score in highest quartile. Low-med. depression: depression *z*-score in lower three quartiles. M, Male; F, Female; dep, Depression *z*-score; med, Intermediate. Additional file 5: Figure S4. Survival by Killip class and beta-blocker use. Survival curves of all subjects (n = 10,512) stratified by Killip class and beta-blocker use adjusted for all other risk factors. BB, Beta-blocker.

## Abbreviations

AUC: Area under the receiving operating characteristic-curve
BDI: Beck Depression Inventory
BMI: Body mass index
HR: Hazard ratio
Lasso: Least absolute shrinkage and selection operator
LVEF: Left ventricular ejection fraction
ZSDS: Zung Self-rating Depression Scale
