# Supplementary material for: Sex dependent risk factors for mortality after myocardial infarction: individual patient data meta-analysis
Source: BMC Med. 2014 Dec 17;12:242. doi: 10.1186/s12916-014-0242-y (PMC4292997; doi:10.1186/s12916-014-0242-y)
Supplement: Additional file 1: Table S1. — Selected interactions in 100 Lasso regression analyses. [file 12916_2014_242_MOESM1_ESM.docx]

**Additional file 1: Table S1. Selected interactions in 100 lasso regression analyses.**

| Selected interactions | Frequency* |
| --- | --- |
| Killip class * beta-blocker use | 95 |
| male sex * LVEF < 40% | 68 |
| male sex * depression high† | 64 |
| male sex * age < 50 | 64 |
| male sex * hyperlipidemia | 61 |
| diabetes * beta-blocker use | 36 |
| hyperlipidemia * Killip class | 34 |
| diabetes * beta-blocker use * depression high† | 31 |
| hyperlipidemia * LVEF < 40% * BMI > 30 | 26 |
| LVEF < 40% * beta-blocker use * BMI > 30 | 20 |
| hyperlipidemia * beta-blocker use | 19 |
| hyperlipidemia * depression low† | 14 |
| diabetes * LVEF < 40% * BMI > 30 | 12 |
| male sex * hyperlipidemia * depression high† | 12 |
| male sex * hyperlipidemia * beta-blocker use | 8 |
| smoking * hyperlipidemia * depression high† | 6 |
| male sex * smoking | 5 |
| male sex * beta-blocker use | 5 |
| LVEF < 40% * beta-blocker use * depression high† | 5 |
| male sex * LVEF < 40% * depression high† | 5 |
| male sex * depression low† | 4 |
| smoking * depression high† | 4 |
| smoking * history of MI * age > 70 | 4 |
| hyperlipidemia * beta-blocker use * depression high† | 4 |
| Killip class * depression low† | 3 |
| male sex * hyperlipidemia * history of MI | 3 |
| LVEF < 40% * BMI > 30 | 2 |
| beta-blocker use * BMI > 30 | 2 |
| beta-blocker use * age > 70 * depression high† | 2 |
| male sex * LVEF < 40% * beta-blocker use | 2 |
| smoking * age < 50 | 1 |
| LVEF < 40% * beta-blocker use | 1 |
| LVEF < 40% * BMI < 20 | 1 |
| history of MI * antidepressant use * age > 70 | 1 |
| LVEF < 40% * Killip class * beta-blocker use | 1 |
| LVEF < 40% * Killip class * BMI > 30 | 1 |
| male sex * LVEF < 40% * antidepressant use | 1 |
| male sex * diabetes * Killip class | 1 |
| male sex * diabetes * beta-blocker use | 1 |
| male sex * beta-blocker use * antidepressant use | 1 |

Abbreviations: BMI, body mass index; LVEF, left ventricular ejection fraction; MI, myocardial infarction.

* The number of times this interaction was found with a penalized beta-coefficient ≥ 0.1 or ≤-0.1 in the models with minimal prediction error resulting from 100 lasso regression analyses in random 80% samples of the training data (n=6,728).

† Depression low and high: depression *z*-score in the lowest and highest quartile. Intermediate depression z-score is the reference class.
