# Supplementary figures and images for: Sex dependent risk factors for mortality after myocardial infarction: individual patient data meta-analysis
Source: BMC Med. 2014 Dec 17;12:242. doi: 10.1186/s12916-014-0242-y (PMC4292997; doi:10.1186/s12916-014-0242-y)

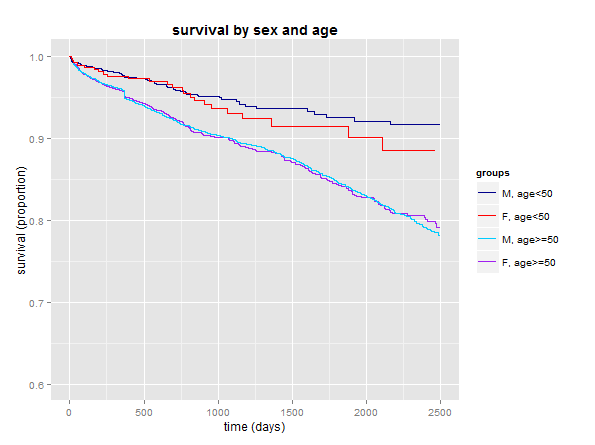

Supplement: Additional file 2: Figure S1. — Survival by sex and age. Survival curves of all subjects (n = 10,512) stratified by sex and age (in years) adjusted for all other risk factors. M, Male; F, Female. [file 12916_2014_242_MOESM2_ESM.png]

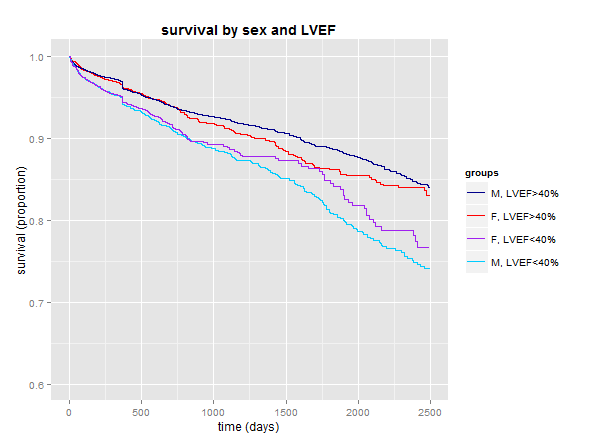

Supplement: Additional file 3: Figure S2. — Survival by sex and left ventricular ejection fraction (LVEF). Survival curves of all subjects (n = 10,512) stratified by sex and LVEF adjusted for all other risk factors. M, Male; F, Female; LVEF, Left ventricular ejection fraction. [file 12916_2014_242_MOESM3_ESM.png]

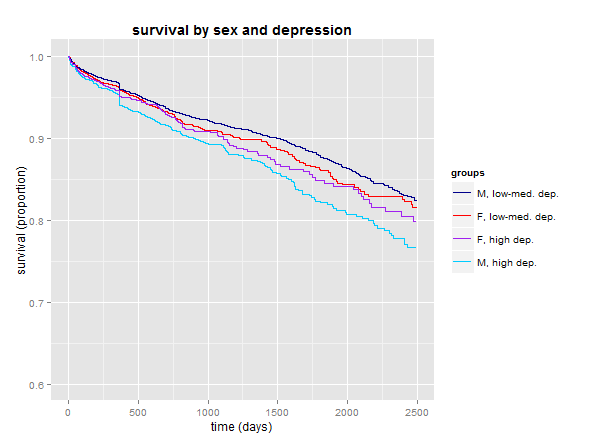

Supplement: Additional file 4: Figure S3. — Survival by sex and depression. Survival curves of all subjects (n = 10,512) stratified by sex and depression z-score adjusted for all other risk factors. High depression: depression z-score in highest quartile. Low-med. depression: depression z-score in lower three quartiles. M, Male; F, Female; dep, Depression z-score; med, Intermediate. [file 12916_2014_242_MOESM4_ESM.png]

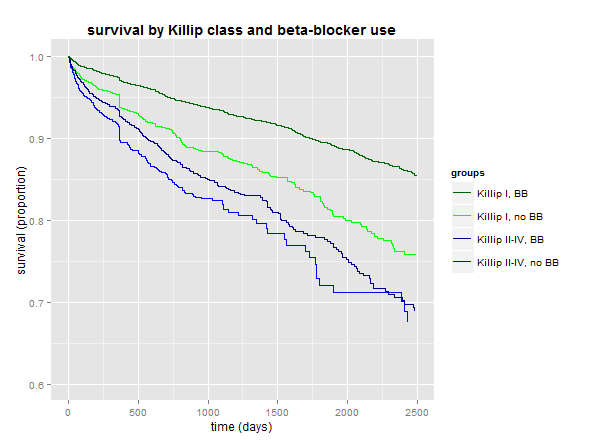

Supplement: Additional file 5: Figure S4. — Survival by Killip class and beta-blocker use. Survival curves of all subjects (n = 10,512) stratified by Killip class and beta-blocker use adjusted for all other risk factors. BB, Beta-blocker. [file 12916_2014_242_MOESM5_ESM.png]
